# Patterns of sexual size dimorphism in horseshoe bats: Testing Rensch’s rule and potential causes

**DOI:** 10.1038/s41598-018-21077-7

**Published:** 2018-02-08

**Authors:** Hui Wu, Tinglei Jiang, Xiaobin Huang, Jiang Feng

**Affiliations:** 1 College of Animal Science and Technology, Jilin Agricultural University, Xincheng ST 2888, Changchun, 130118 China; 2 Jilin Provincal Key Laboratory of Animal Resource Conservation and Utilization, Northeast Normal University, Jingyue St 2555, Changchun, 130117 China; 3 Key Laboratory of Vegetation Ecology of Education Ministry, Institute of Grassland Science, Northeast Normal University, Changchun, 130024 China

## Abstract

Rensch’s rule, stating that sexual size dimorphism (SSD) becomes more evident and male-biased with increasing body size, has been well supported for taxa that exhibit male-biased SSD. Bats, primarily having female-biased SSD, have so far been tested for whether SSD allometry conforms to Rensch’s rule in only three studies. However, these studies did not consider phylogeny, and thus the mechanisms underlying SSD variations in bats remain unclear. Thus, the present study reviewed published and original data, including body size, baculum size, and habitat types in 45 bats of the family Rhinolophidae to determine whether horseshoe bats follow Rensch’s rule using a phylogenetic comparative framework. We also investigated the potential effect of postcopulatory sexual selection and habitat type on SSD. Our findings indicated that Rensch’s rule did not apply to Rhinolophidae, suggesting that SSD did not significantly vary with increasing size. This pattern may be attributable interactions between weak sexual selection to male body size and strong fecundity selection for on female body size. The degree of SSD among horseshoe bats may be attributed to a phylogenetic effect rather than to the intersexual competition for food or to baculum length. Interestingly, we observed that species in open habitats exhibited greater SSD than those in dense forests, suggesting that habitat types may be associated with variations in SSD in horseshoe bats.

## Introduction

Animal body size is an important trait under evolutionary scrutiny^1^. For small mammals such as rodents and bats, body size plays a pivotal role in shaping variations in reproductive success^2,3^. Body size dimorphism between sexes is ubiquitous in nature and extensively varies even among closely related groups. Rensch (1950, 1960) was the first to observe a common pattern of interspecific covariance between body size and sexual size dimorphism (SSD)^4^. Rensch’s rule predicts that when males are larger than females, SSD increases with body size, but when females are larger than males, SSD decreases in larger species. Rensch’s rule implies that male body size has a faster evolutionary rate than females^5,6^. Rensch’s rule was originally formulated at the interspecific level, and most publications on SSD present broad interspecific comparisons. For example, there is strong support for Rensch’s rule in all mammals and birds^7^. Rensch’s rule has been confirmed at the macroecological level by observations in various animal taxa including insects^8,9^, fish^10^, reptiles^11^, birds^12,13^, and primates^14^. In general, SSD allometry consistent with Rensch’s rule occurs most frequently in animals for which males are larger than females. Previous studies on SSD in taxa that display female-biased SSD provide mixed support for Rensch’s rule^15–18^.

Most mammals exhibit SSD and is mainly male-biased. Bats, however, primarily display female-biased SSD^19^. Previous studies have shown that various species of Vespertilionidae, Rhinolophidae, and Pteropodidae have female-biased SSD with respect to body mass and skeletal measures (e.g., forearm length), but these studies have mostly focused on SSD differences both within and between species^20–23^. Little is known about whether bats follow Rensch’s rule at the intraspecific or interspecific level and the causes for different SSDs in the context of a phylogenetic comparative framework. To date, three studies have tested Rensch’s rule in bats. One study confirmed that the pattern of SSD in *Myotis* bats did not conform to Rensch’s rule^24^. The second study indicated that variations in SSD among greater horseshoe bat (*Rhinolophus ferrumequinum*) populations is consistent with Rensch’s rule^25^. The third study showed that variations in body size in 10 bat species (2 from Vespertilionidae and 6 from Phyllostomidae) apparently do not conform to Rensch’s rule at intraspecific and interspecific levels^26^. Therefore, additional studies validating Rensch’s rule in bats (a unique mammal group), especially after controlling for phylogenetic associations, are warranted.

Biologists generally explain SSD in terms of sexual selection^27–29^. SSD is often used as an indicator of the intensity of precopulatory sexual selection^30,31^. Meanwhile, sexual selection is to drive diversity of baculum form^32,33^. Large baculum and testes are expected to confer an advantage in sperm competition and reproductive success^34^. Baculum size (adjusted for body size) has often been used as a proxy of the intensity of postcopulatory sexual selection. Beacuse large testes/bacula and increased spermatogenesis entail high production cost and is hypothesized to be associated with a trade-off between pre- and postcopulatory traits^35^. For example, a trade-off (negative correlation) between precopulatory (SSD) and postcopulatory (relative testes mass/baculum length) traits was observed in 14 species of pinnipeds^36^ and in 17 male cetaceans^35^.

In addition to sexual selection, habitat type is also an important ecological factor that influences SSD. Species living in different habitats may need to deal with variations in environmental conditions (e.g., food availability, competitors and predators), any of which could be associated with SSD variations among species^37^. For example, the existence of habitat-specific sexual dimorphism has been reported in *Anolis* spp.^38^. Moreover, males in 138 turtle species are proportionally smaller in more aquatic habitats and larger in terrestrial habitats^39^.

**Figure 1:**
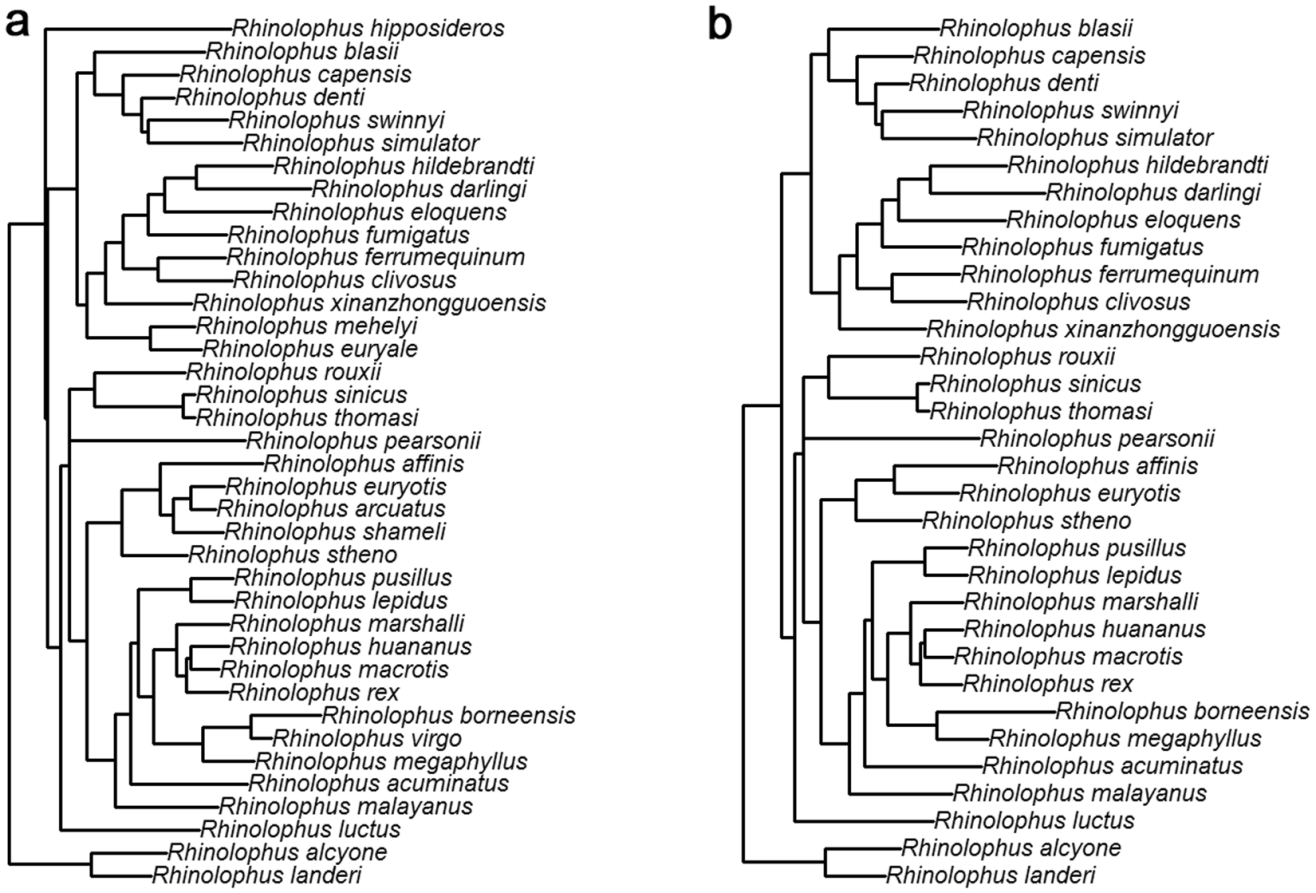
Maximum likelihood phylogeny tree of horseshoe bats. (**a**) species for which there are forearm length data (N = 38), (**b**) species for which there are body mass data (N = 32).

Rhinolophidae is a suitable clade for studying body size patterns change and associated mechanisms as it is the second most speciose bat species (77 species listed in^40^). Linear forearm length ranges from 30 cm in adult *Rhinolophus subbadius* to 75 cm long in *R*. *luctus*, and this genus exhibits predominantly female-biased size dimorphism, an uncommon pattern in other mammalian taxa. Additionally, they are ecologically diverse, inhabit different habitats across temperate and tropical regions of the Old World^41,42^. Although horseshoe bats all fly close to the substrate and vegetation irrespective of habitat type or their body size^43^, some species prefer relatively open habitats for foraging such as arid areas, fynbos (a small belt of natural shrubland or heathland vegetation), and savanna woodlands, whereas others occupy ecosystems with dense vegetation (e.g., forests). For example, *R*. *mehelyi* always forages in woodlands and avoids open spaces, suggesting that this species prefers cluttered spaces^44^. In the present study, we analysed a comprehensive dataset of male and female body mass and forearm length estimates for 45 species of horseshoe bats using a unifying comparative phylogenetic framework (Tables 1 and 2; Fig. 1). Our aims were: (1) to test whether patterns of interspecific variations in SSD in horseshoe bats conform to Rensch’s rule, (2) to test the hypothesis that habitat types fuel variations in SSD among horseshoe bats, and (3) to test the additional alternative hypothesis that differences in SSD are the result of sexual selection by assessing the relationship between baculum size and SSD or are simply a reflection of phylogenetic constraint.

**Table 1:** Sample sizes for body size data and all other data used in this study.

Traits	Number of species	Kolmogorov-Smirnov test for males	Kolmogorov-Smirnov test for females
forearm length	45	*P* > 0.05	*P* > 0.05
body mass	34	*P* > 0.05	*P* > 0.05
forearm length n ≥ 5	33	*P* > 0.05	*P* > 0.05
body mass n ≥ 5	21	*P* > 0.05	*P* > 0.05
cytochrome b (cytb)	38		
Baculum length	21	*P* > 0.05	*P* > 0.05
Baculum width	15	*P* > 0.05	*P* > 0.05
habitat	30

**Table 2:** Body, baculum size, and habitat type data from studies of horseshoe bat species included in our analyses. Open habitats represent fynbos, arid areas, savanna woodland, hedgerows, riparian forest, pastures, hedges, and ditches.

*Species*	Location and Time	Male		Female		Male		Female		Baculum		Cytb GenBank Accession #	Habitat type	Ref.
		Forearm (mm)	N	Forearm (mm)	N1	Body mass	N2	Body mass	N3	Length	Width			
*R*. *maendeleo*	Tanzania, Africa (5.08°S, 39.03°E; 1985, 1992)	47.2	1	48.3	1	6	1	—	—	3.34	1.07		Forest	^42,91^
*R*. *capensis*	Extreme southwest of Africa (28°–34°S, 16°–28°E)	49.3	11	50.2	5	10.5	7	12.9	4	—	—	FJ185190	Open (Fynbos and succulent karoo biomes)	^72^
*R*. *denti*	Southern Africa (16°–32°S, 12°–26°E)	42.7	13	43.5	14	6.5	13	7.4	13	—	—	FJ185193	Open (Arid habitats)	^72^
*R*. *simulator*	East parts of southern and central Africa (12°–32°S, 24°–38°E)	44.4	33	45.1	37	7.3	17	9.98	18	—	—	EU436670	Open (Savanna woodland)	^72^
*R*. *swinnyi*	East parts of southern and central Africa (12°–33°S, 26°–37°E)	41.7	23	42.5	14	6.6	12	7.2	3	—	—	FJ185214	Open (Savanna woodland)	^72^
*R*. *euryale*	Southeastern Europe (Bulgaria, Greece, and Turkey; 1999–2004)	47.4	399	47.9	512	—	—	—	—	3.25	—	EU436671	Open (Savanna woodland/hedgerows and woodland edges))	^20,92^
*R*. *mehelyi*	Europe (Iberia, France, Italy, Greece, Romania; 1999–2004)	51.1	218	51.5	548	—	—	—	—	2.8	—	EU436672	Forest (Between grass stems or bush edges)	^20,44,93^
*R*. *arcuatus*	Tentena Poso, Sulawesi, Indonesia (1.37°S, 120.74°E; 1987)	49.8	1	50.5	6	—	—	—	—	—	—	JN106301	Open (Forest/Savanna)	^94^
*R*. *coelophyllus*	Southeast Asia (Myanmar, Thailand, Malaysia; 2006–2008)	44.3	26	44.3	9	—	—	—	—	—	—		Forest (hill evergreen and deciduous forest, agricultural land)	^95^
*R*. *euryotis*	New Guinea (YUS Conservation Area; 5.99°S, 146.86°E)	56.97	12	56.98	48	18.04	12	18.52	48	—	—	JN106276		^96^
*R*. *shameli*	Southeast Asia (Thailand, Cambodia, Vietnam; 2006–2008)	46.5 ^M^	12	46	8	—	—	—	—	—	—	JN106269	Forest (deciduous and evergreen forest)	^95^
*R*. *clivosus*	East parts of southern Africa (8°–34°S, 16°–36°E)	53.1	86	54.1	59	16.2	40	18.8	32	3.06	—	FJ185191	Open (Savanna woodland, Riparian forest)	^72^
*R*. *darlingi*	Southern Africa (12°–32°S, 12°–36°E)	46.3	40	49.1	19	8.8	25	12.2	10	—	—	FJ185192	Open (Savanna woodland)	^72^
*R*. *ferrumequinum*	Southeastern Europe China (Ji’an; 41.05°N, 125.83°E)	57	117	58.3	1010	17.88	31	22.69	77	3.7	0.9	AB085731	Open (Savanna woodland/Pastures/Hedges)	^20,93^
*R*. *eloquens*	Southern Africa	58.1	122	58.7	108	21	104	21.5	102	—	—	EU436677		^72^
*R*. *fumigatus*	Southern and central Africa (8°–24°S, 12°–36°E)	53.5 ^M^	15	52.7	6	13.7	7	14.2	1	—	—	EU436678	Open (Arid savanna, savanna woodland)	^72^
*R*. *hildebrandtii*	Northeast of southern and central Africa (8°–26°S, 24°–40°E)	63.4	17	63.5	18	23.4	7	27.2	11	—	—	EU436676	Open (Savanna woodland)	^72^
*R*. *hipposideros*	Southeastern Europe (Bulgaria, Greece, and Turkey; 1999–2004)	37.2	30	38.5	18	—	—	—	—	3.31	—	KC579369	Open (Herbaceous vegetation/Ditches/Hedges)	^20^
*R*. *alcyone*	Africa (From Uganda and Sudan to Guinea and Senegal)	53 ^M^	39	52.9	39	15.6 ^M^	32	14.4	31	—	—	FJ457613	Forest	^72^
*R*. *blasii*	Southeastern Europe; southern Africa (12°–32°S, 26°–38°E)	46.2	60	47.2	169	8	6	10	3	2.13	—	FJ185188	Open (Savanna woodland and montane)	^20,72,97^
*R*.*xinzhongguoensis*	China (Yongde, Yunnan (24.36°N, 99.65°E); Suiyang, Guizhou (28.22°N, 107.15°E); 2005)	59.6	3	60.2	2	24 ^M^	2	21.5	2	—	—	EU391626		^98^
*R*. *landeri*	Northeast of southern and central Africa (12°–24°S, 28°–40°E)	44.3 ^M^	7	43.7	8	8	2	8.2	7	—	—	FJ457612	Open (Forest and riparian woodland)	^72^
*R*. *affinis*	China (Yunnan; 24.50°N, 102.34°E; 2006–2007)	50.96	34	51.88	28	11.19	34	11.59	28	2.08	0.67	EF544420	Forest (subtropical secondary forest)	U
*R*. *borneensis*	Male (Cambodian; 11.92°N, 106°E), Female (Batu Punggul, Malaysia; 4.63°N, 116.62°E)	44.7 ^M^	2	43.63	8	9.45	2	10.88	8	—	—	EU521608	Forest (rainforests)	^99,100^
*R*. *malayanus*	Myanmar (Mon, Kayin and Shan States; 1999–2003)	41.3	6	41.4	6	6.8 ^M^	6	6.3	6	—	—	FJ185205	Forest (coastal rain forest and moist deciduous forest)	^101^
*R*. *megaphyllus*	New Guinea (YUS Conservation Area; 5.99°S, 146.86°E)	48.44	3	49.82	10	10.33	3	10.42	10	5		FJ185207	Forest	^66,96^
*R*. *stheno*	Southeast Asia (Chiang Mai, Tak, Loei and Petchabun province)	45.2	21	45.2	14	7.8 ^M^	18	7.6	12	—	—	FJ185213	Forest (hill evergreen forest and mixed deciduous forest)	^102^
*R*. *virgo*	Philippines (Palawan Island)	40.5	3	41.5	3	—	—	—	—	—	—	JN106309	Forest (secondary, primary lowland and mossy forest)	^103^
*R*. *pearsonii*	China (Jiangxi; 26.60°N, 114.21°E; 2006, 2009)	53.38	28	54.42	18	13.63	28	15.77	18	2.89	1.11	JX502551	Forest (bamboo forest and mixed forest)	U
*R*. *yunanensis*	Myanmar (Mon State; 16.37°N, 97.77°E)	55	2	56.8	2	—	—	—	—	—	—	Y		101
*R*. *macrotis*	China (Yunnan; 24.50°N, 102.34°E;2007)	42.52	19	46.72	13	7.05 ^M^	19	6.9	13	3.82	0.86	JX465355	Forest (subtropical secondary forest)	U
*R*. *marshalli*	China (Yunnan (22.61°N, 103.85°E; 2009); Guangxi (41.01°N, 125.85°E; 2009))	44.38	12	45.31	15	6.16	12	7.31	15	3.7	1.05	EU434938	Forest (secondary and mixed forest)	U
*R*. *rex*	China (Guizhou, 27.99°N, 107.17°E; 2008)	55.17	12	56.82	6	9.75	12	10.66	6	4.84	1.71	EU075216	Forest (secondary and mixed forest)	U
*R*. *huanus*	China (Jiangxi; 26.60°N, 114.21°E; 2006)	39.13	5	39.74	9	4.45	5	4.67	9	3.65	1.14		Forest (bamboo forest and mixed forest)	U
*R*. *acuminatus*	Vietnam (Tay Ninh, Cat Loc and Ma Da province)	48.8 ^M^	3	46.8	3	12.5 ^M^	3	9.9	3	—	—	EF108155	Forest (lowland dipterocarp forests)	^104^
*R*. *pumilus*	Japan (Okinawa-jima Island; 2003–2004)	39.1	24	39.9	56	—	—	—	—	—	—			^105^
*R*. *lepidus*	China (Yunnan; 24.50°N, 102.34°E; 2006–2007)	42.41	65	42.99	22	6.56	65	6.89	20	4.11	1.08	FJ185202	Forest (subtropical secondary forest)	U
*R*. *osgoodi*	China (Yunnan; 24.50°N, 102.34°E; 2008)	41.36	11	44.72	2	5.71	11	—	—	4.06	0.73	Y	Forest (subtropical secondary forest)	U
*R*. *shortridgei*	Myanmar (Gwa Township, Pyay and Kanbalu)	40.1 ^M^	8	39.2	5	6.8 ^M^	1	6.2	1	—	—			^106^
*R*. *pusillus*	China (Hubei, 30.71°N, 115.73°E; 2010)	37.23	48	38.22	61	3.65	7	4.17	9	4.39	1.3	EF544425	Forest (secondary forest and agricultural field)	U
*R*. *subbadius*	Myanmar (Nam Tamai Valley, Kachin State)	33.9	5	35.05	2	16	5	17.5	2	—	—	Y		^101^
*R*. *rouxii*	Vietnam	44.4 ^M^	3	43.9	7	8.5	3	9.9	7	2.3	0.7	JQ316214	Forest	^104,107^
*R*. *sinicus*	China (Hunan, 27.74°N, 117.70°E; 2010)	45.87	25	45.9	19	10.08	25	10.53	19	2.17	0.61	HM134917	Forest (secondary forest and agricultural field)	U
*R*. *thomasi*	China (Jiangxi; 29.38°N, 117.70°E; 2009)	44.66	21	44.83	18	8.99	21	9.12	18	1.99	0.55	EU434943	Forest (secondary forest)	U
*R*. *luctus*	China (Hainan; 18.71°N, 108.87°E; 2008)	63.27	1	67.77	3	22.39	1	27.81	3	6.8	2.47	EU521609	Forest (evergreen and mixed deciduous forest)	U

U: Unpublished Data. The superscript letters “M” represents males in forearm length and/or body mass were larger than females (male-biased SSD).

## Results

**Figure 2:**
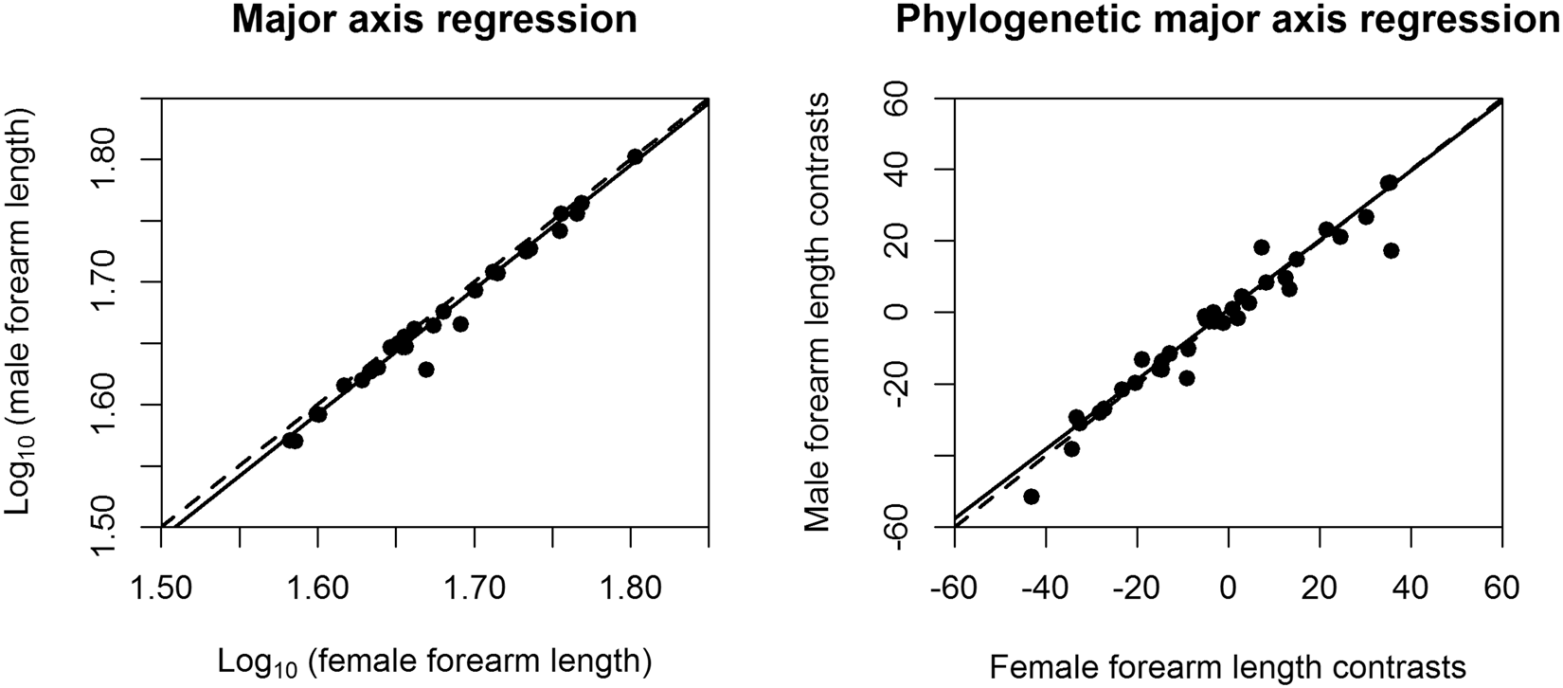
Rensch’s Rule for horseshoe bats using forearm length with phylogenetically uncorrected and corrected conditions. Black lines, major-axis regression line; black dashed lines, slope = 1.

**Figure 3:**
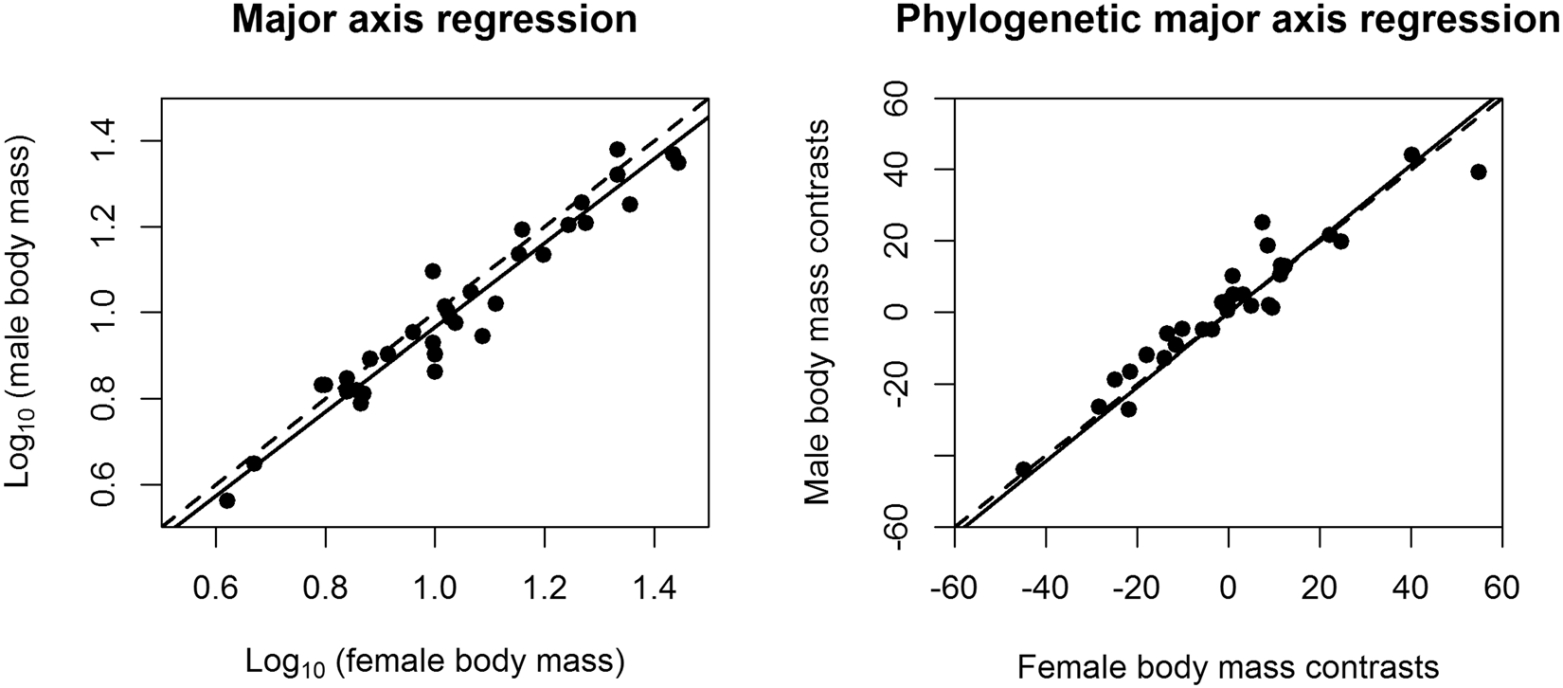
Rensch’s Rule for horseshoe bats using body mass for phylogenetically uncorrected and corrected conditions. Black lines, major-axis regression line; black dashed lines, slope = 1.

The taxa exhibited varying degrees and directions of size dimorphism. In 37 out of 45 species of horseshoe bats, females showed longer forearms than males. In 27 out of 34 species of horseshoe bats with body mass data, females were larger in twenty-seven of them (see Table 2 for details). On average, SSD in Rhinolophdae is female-biased. The reduced major axis regression of log_10_ (male size) on log_10_ (female size) showed that the allometric slopes did not significantly differ from 1. After correcting for phylogeny, the slopes still did not significantly differ from 1 (Table 3; Figs 2 and 3). After excluding species with male-biased SSD and fewer than five individuals of each sex, we obtained the same results, i.e., an allometric slope that did not differ from 1, and SSD following an isometric pattern.

**Table 3:** Major-axis regression results of male size on female size (log_10_-transformed) for uncorrected data and for phylogenetically independent contrasts (PICs).

Trait	N	N_FSSD_	N_MSSD_	Phylogenetically uncorrected	Phylogenetically corrected	Different from 1 or not	*P*	Rensch’s rule?
				MA Slope (95% CI)	PMA Slope			
Forearm length	45	37	8	0.997(0.994,1.052)	0.973	NOT	0.91	NOT
Body mass	34	27	7	0.98(0.892,1.077)	1.034	NOT	0.67	NOT

### Phylogenetic analyses

SSD in body mass and body mass of both sexes were significantly influenced by phylogeny (SSD: λ = 0.999, *p* < 0.001; K = 0.764, *P* = 0.028; male body mass: λ = 0.999, *P* = 0.002; K = 0.901, *P* = 0.01; female body mass: λ = 0.999, *P* = 0.0005; K = 0.976, *P* = 0.004), whereas both female and male forearm length showed a weaker phylogenetic signal (male forearm length: λ = 0.494, *P* = 0.309; K = 0.629, *P* = 0.083, female forearm length: λ = 0.455, *P* = 0.316; K = 0.612, *P* = 0.097), suggesting that related species were not statistically more likely to have similar forearm length than would be expected.

**Figure 4:**
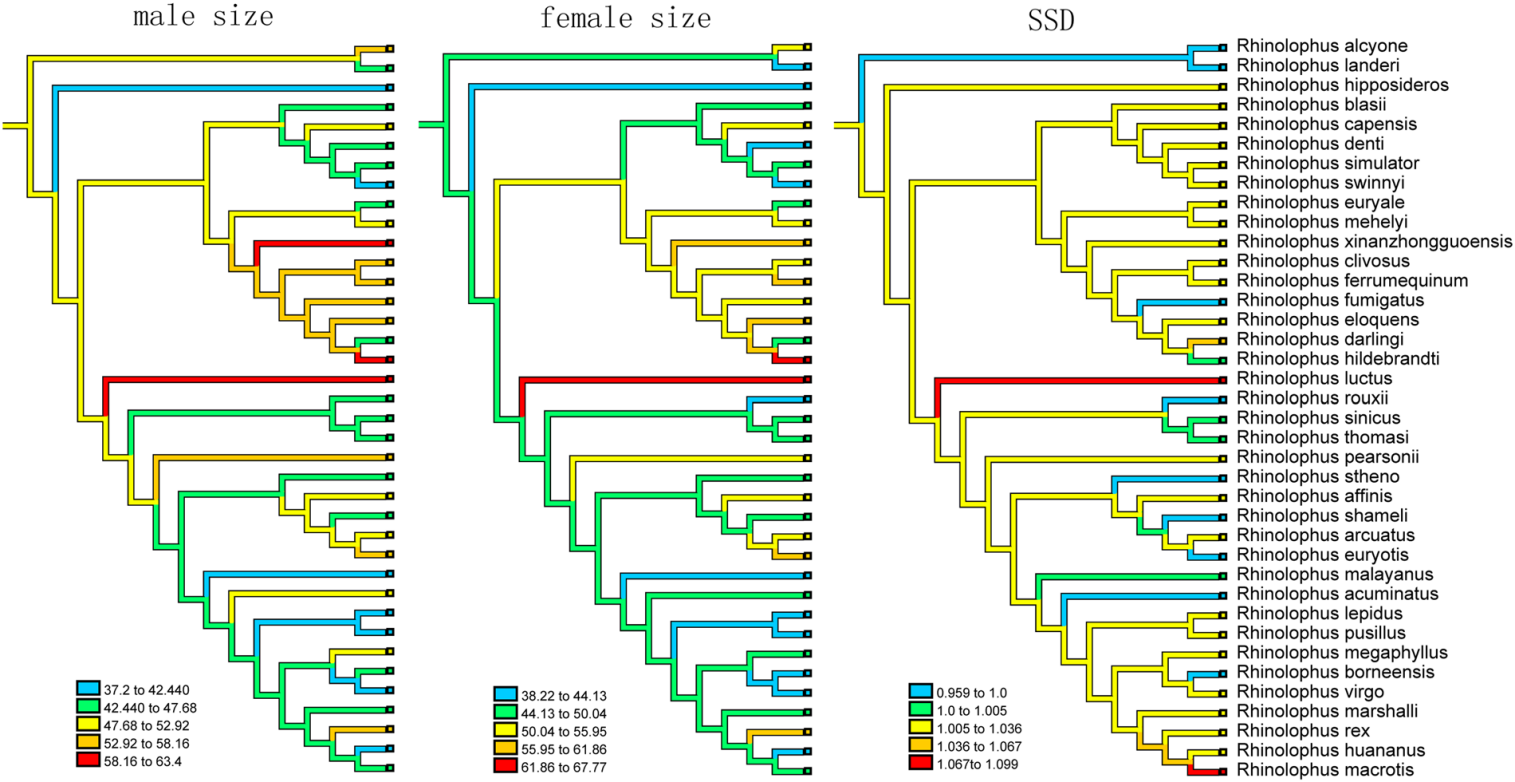
Reconstructed evolution of male forearm length (mm), female forearm length (mm), and SSD for the best ML tree. Colors denote size classes (legends).

Tracing the evolution of body size and SSD on a pruned ML phylogeny unequivocally optimized median size (male forearm length = 48.37 mm; female forearm length = 48.88 mm), and female-biased SSD (SD = 1.009) was observed as ancestral in Rhinolophidae (Fig. 4). Size evolution was then inferred to have proceeded through repeated increases and decreases, and SSDs were variable among species (Fig. 4).

### The influence of habitat type on the evolution of SSD

**Figure 5:**
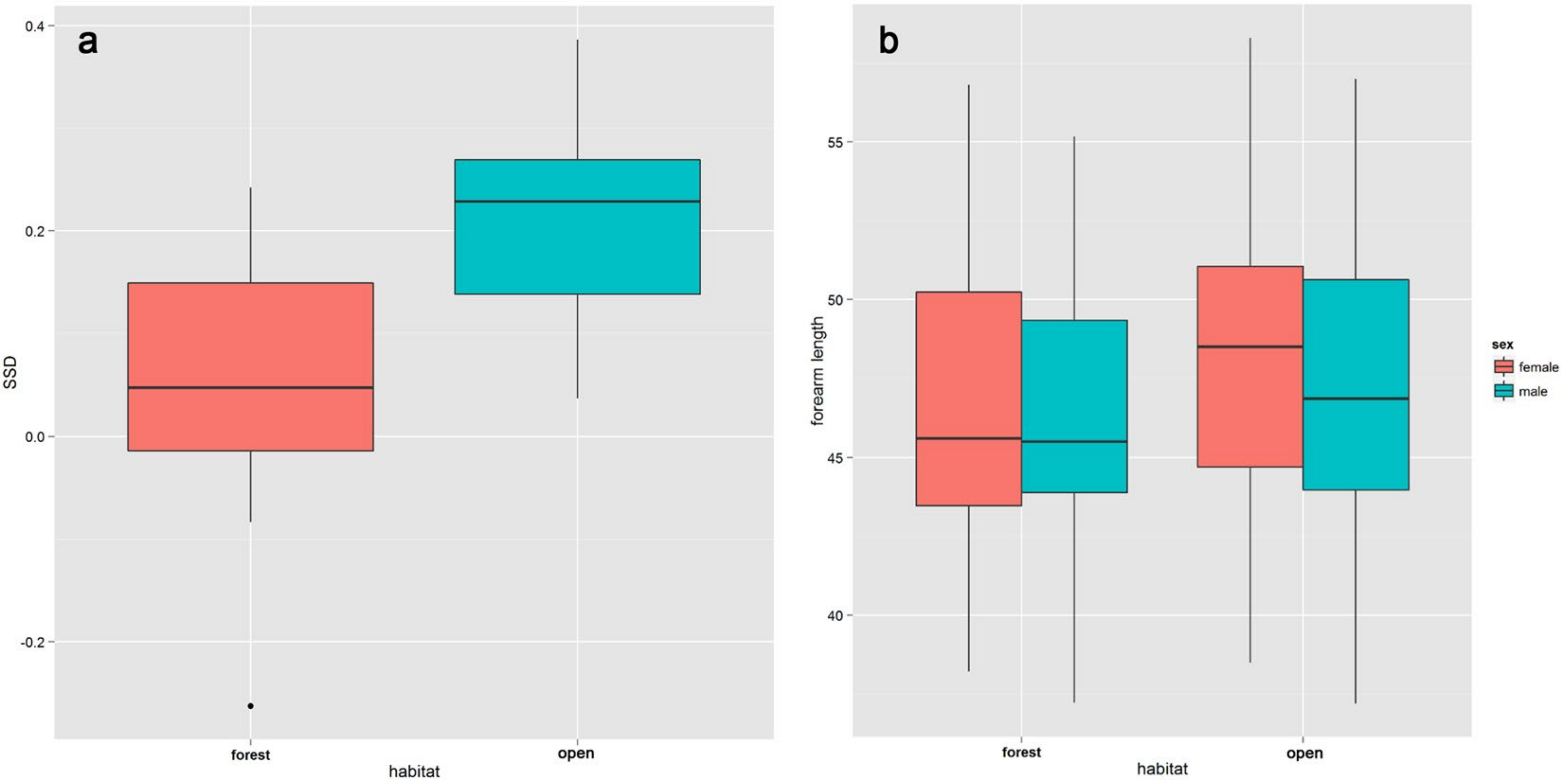
Difference in mean SSD between species inhabiting the forest (closed habitat) and in other (open) habitats. (**a**) SSD between habitat types, (**b**) forearm length between sexes in different habitat types.

SSD significantly differed between open and closed habitat lineages when phylogeny was not considered (ANOVA: *F*_*1*_,_2*5*_ = 11.47, *P* = 0.002). Species inhabiting open habitats showed greater mean dimorphism than those linked to forest habitats (Fig. 5a). Additionally, female forearm length was greater than that in males in open habitats compared to those in forests (Fig. 5b). After using independent contrasts to control for phylogenetic inertia in these data, SSD in forearm length did not correlate with habitat types (phyaov: *F*_*1*_,_2*5*_ = 11.47, *P* = 0.15).

### The influence of baculum size on the evolution of SSD

Linear regression demonstrated that male body size is not associated with baculum length (forearm length: *R*^*2*^ = 0.09, *F*_*1*,*19*_ = 1.904, *P* = 0.184; body mass: *R*^*2*^ = 0.10, *F*_*1*,*14*_ = 1.539, *P* = 0.235). After using independent contrasts to control for phylogenetic inertia in these data, male forearm length was positively associated with baculum length (pgls: *F*_*1*,*17*_ = 6.263, *R*^*2*^ = 0.269, *P* = 0.005). Additionally, our results showed that SSD is positively associated with baculum length (*R*^*2*^ = 0.291, *F*_*1*,*18*_ = 7.382, *P* = 0.014). However, when controlling for the effect of male body size, SSD was not significantly associated with baculum length (male forearm length × baculum length: *R*^*2*^ = 0.323, *F*_*3*,*16*_ = 0.0919, *P* = 0.649). Thus, our study found no clear relationship between baculum size and dimorphism.

## Discussion

In this study, we found that the slopes of the regression of log_10_ (male size) on log_10_ (female size) were not significantly greater than one regardless of traits of body size and data sets, suggesting no evidence to support Rensch’s rule among horseshoe bats (Table 3). Moreover, our results indicated that species occupying open habitats had greater mean dimorphism compared to those in forest habitats, suggesting that habitat type may drive the evolution of SSD among horseshoe bats. Additionally, SSD was not significantly associated with baculum length when controlling for the effect of male body size, suggesting that baculum length may not be linked with the degree of SSD among horseshoe bats.

As small mammals, horseshoe bats have relatively subtle size differences between males and females. Our results indicated that SSD patterns in horseshoe bats do not conform to Rensch’s rule irrespective of SSD measurements (body mass *vs*. forearm length) and computation method (conventional *vs*. phylogenetically informed). This study joins other gruops that question the veracity of Rensch’s rule^15,45–47^, especially in taxa with female-biased SSD. Similarly, our results agree with previous work on SSD in bats, as one recent study showed that *Myotis* does not follow Rensch’s rule among species either^24^. Another study including seven phyllostomid and two *Myotis* bat species also reported similar results^26^. However, variations in SSD among populations within *R*. *ferrumequinum* conform to Rensch’s rule^25^. These studies show inconsistencies in SSD variations in horseshoe bats between interspecific and intraspecific comparative analysis. Additionally, the present study observed that the most recent common ancestor in Rhinolophidae exhibited medium-sized forearm length (males = 48.37 mm; females = 48.88 mm, Fig. 4), similar to that of a previous study (forearm length: 50.16)^48^, and also matched well with the size observed in many median-size horseshoe bats^42^.

Several factors may explain the lack of conformity to Rensch’s rule and female-biased SSD in horseshoe bats. First, the limited extent of SSD in this group reduces statistical power to detect any existing trend. Second, because horseshoe bats possess extensive variability with respect to mating systems (e.g., polygamy in *R*. *ferrumequinum*^49^ *vs* monogamy in *R*. *luctus*^50^), sexual selection for male size may be nondirectional. Third, sexual selection may act more on echolocation call frequencies in males than body size in horseshoe bats, suggesting weak sexual selection to body size in males^48,51^. Meanwhile, in bats, fecundity selection might confer advantages to larger female body size, such as reduced proportionate fetal or newborn load^52^, increased stomach capacity for food, reproductive success, and reduced relative cost of milk production^22,53^. In this case, interactions between weak sexual selection in males and strong fecundity selection in females are likely to determine female-biased SSD in horseshoe bats, and thus may explain the observed nonconformity to Rensch’s rule because stronger sexual selection in males in conjunction with weaker selection in females is considered a major determinant of Rensch’s rule (sexual selection is for increased male size in species with male-biased SSD when that trait results in greater mating success; sexual selection is for reduced male size in taxa with female-biased SSD)^12^.

Although habitat has been suggested to influence the evolution of SSD, this hypothesis has rarely been demonstrated. In this study, we found that species in open habitats (e.g., fynbos, savanna woodland, and arid) exhibit more extensive SSD than those inhabiting highly dense forests (Fig. 5a). The role of SSD in reducing competition between sexes is often assessed in investigations that aim to identify habitat-related factors that fuel SSD evolution. The niche theory predicts that SSD should be smaller when the range of available resources is smaller. The large SSD may be selected for by intraspecific competition when different sizes deemed most effective among various resource types^54^. A few studies have validated the assumption that SSD reduces intersexual competition^38^. Dechmann *et al*.^55^ did not observe any difference between male and female diets in the common noctule (*Nyctalus noctula*). In horseshoe bats, echolocation call frequencies of females in most species are only slighter higher than that of males, and such small differences seem inappropriate to differentiate prey size. In this case, sex differences in body size may not reduce competition between the sexes for food resources in both open and forest habitats.

Selective pressures might influence SSD as it relates to structural habitat^38^. Optimal feeding models predict that the distribution of optimal body size may depend on forage strategy^56^, i.e., active searchers display unimodal plots, whereas sit-and-wait predators have bimodal plots. Thus, SSD should be more distinct among the latter. This prediction has been validated in greater Antillean *Anolis* lizards^38^. Two foraging styles, aerial hawking and flycatching, are often used by horseshoe bats to hunt for insects^57,58^. A previous study showed that horseshoe bats perform flycatching (a sit-and-wait strategy) more than aerial hawking to save energy when there are relatively fewer insects^59^. Tree density and diversity in open habitats (e.g., fynbos, savanna woodland, and arid in Africa) are lower than in tropical moist forest biomes (e.g., Southeast Asia and southern China)^60^. Because diversity and richness of plants are useful proxies for insect abundance^61^, the present study presumed that insect richness in open habitats would be lower than that in thick forests. Thus, in open habitats, flycatching would be mainly employed to hunt prey and would be associated with greater SSD, whereas those inhabiting dense forests may mainly be active searchers and have a lower degree of SSD.

In this study, females in open habitats had larger forearms than males compared to that in forests (Fig. 5b), which in turn may lead to bigger SSD in open habitats. In the horseshoe bat, sexual segregation is common after mating in the spring. Females and their offspring often constitute a maternal colony^58^. In open habitats, the distribution of food resources is relatively disperse. In this case, the intensity of competition for food among females may be relatively small, which may increase the number of larger females in open habitats compared to the forest. Moreover, females in open habitats have to spend more time foraging compared to their forest counterparts because of food dispersion^49,57^. To save energy, they evolved larger bodies to reduce the surface-to-volume ratio for heat conduction. Interestingly, SSD did not correlate significantly with habitat types after using independent contrasts to control for phylogenetic inertia, suggesting that differences in SSD among horseshoe bats may be attributed to their evolutionary history (common ancestor) rather than to adaptation to different habitat types. However, we should be cautious because only two types of habitats were studied here, and this may suggest that closely related species may have the same habitat types. This would hence magnify the effect of phylogeny during analysis. Thus, further experimental examination will help to clarify the relationship between SSD and habitat types in bats.

No clear relationship between baculum size and SSD was observed in the present study, although SSD appeared to be positively associated with baculum length. However, when controlling for the effect of male body size, SSD was not significantly associated with relative baculum length. Similar to other related studies in mammals^62,63^, we found no evidence of a trade-off between precopulatory (SSD) and postcopulatory (baculum length) traits in horseshoe bats. In fact, a few studies suggest a theoretical trade-off between pre- and postcopulatory traits across a phylogeny, perhaps due to complications of interspecific comparisons. The correlation between postcopulatory traits (testes mass and/or baculum length) and SSD exsits only in polygamous species for which males engage in competition to monopolize access to multiple females^64^. This is unlikely to be the case for all horseshoe bats, as polygamy is not a universal trait of all horseshoe bat species (e.g., monogamous *R*. *luctus*). Furthermore, bats are the only mammals that truly fly, thus traits in bats favored by sexual selective pressure may be differ from those of other mammals. For example, female greater sac-winged bats (*Saccopteryx bilineata*) favor small and symmetric males for mating^65^. Moreover, female in *R*. *mehelyi* preferentially select males with high frequency echolocation calls for mating^51^. Additionally, bat baculum length may not to be associated with sexual selection intensity according to comparative analysis^66^. These studies indicated that sexual selection pressure on male bats may preferentially act on smaller body size and higher echolocation call frequencies rather than larger body size or baculum size. Thus, these possibilities may obscure a clear prediction of the trade-off hypothesis on precopulatory versus postcopulatory investment, as well as suggest that sexual selection may not be associated with variations in SSD among horseshoe bats. Future studies should thus aim to generate direct evidence for the relationship between sexual selection and SSD in future studies.

Some studies have suggested that phylogenetic constraints are mainly responsible for the degree of sexual dimorphism^67^, and the findings of the present study support this theory. We found a significant phylogenetic signal with regard to body mass of the two sexes and SSD, suggesting that closely related species should exhibit highly in SSD than more distantly related ones because they share more recent ancestors. Additionally, repeated increases and decreases in SSD among horseshoe bats were noted during evolution.

## Conclusion

In summary, we found that SSD variations among species within the Rhinolophidae do not conform to Rensch’s rule. These results are similar to the findings of previous studies done involving Vespertilionidae and Phyllostomatidae, suggesting that Rensch’s rule may not be applicable to all bat species within a family. Despite extensive efforts in identifying the causal mechanisms of SSD evolution, only a few studies have investigated the impact of ecological factors. Our results indicate that evolutionary changes in the degree of SSD among horseshoe bats may undergo phylogenetic constraints, and that the evolotuin of SSD may be closely linked with habitat types rather than sexual selection. A critical limitation of this study is that the results relating to SSD and habitat types should be interpreted with caution because habitat types of horseshoe bats have to be classified as either open or forest in the absence of specific habitat use information in each species. Future studies should focus the relationship between the degree of SSD and habitat use in sympatric horseshoe bats.

## Materials and Methods

### Taxa Sampling

Morphometric data (body mass, forearm length, baculum length, and baculum width) were collected from published and our unpublished data (Table 1). We carefully analyzed the literature to collect individual data. For our own data, every individual was only measured once. Data from 45 species of horseshoe bats were collected in this study. Twenty species had exact coordinate information about the sample sites (Table 2). Ten species collected in southern and central Africa from the literatures only had the distribution range for the sample sites (Table 2). For the other 15 species, since their data was collected from a area including several neighboring sites, exact coordinate information and sample size for every site was not available in the literatures (Table 2). In this case, all individual data from different sites of a species was pooled to calculate mean value for subsequent analyses. Although the presence and level of SSD may depend on the sample sites and/or populations in some phyllostomid bats^26^, this was not this case in horseshoe bats for two reasons. First, SSD was observed for most horseshoe bats in this study (see Table 2 for details). Second, in our previous study^25^, we analyzed SSD of 23 populations of *R*. *ferrumequinum* with a wide range of distributions, and found that SSD was consistently female-biased and not statistically significantly different among along a latitudinal cline, suggesting environmental conditions may not influence in SSD variation at intraspecific level. These two facts implied that SSD in horseshoe bats may be only slightly different among sites or populations. Additionally, although individuals of some species were captured at different times, this did not influence our results because only adult data was collected in this study. In this case, it may be rational to pool data of a species from different sites or times to increase sample size, especially for interspecific comparative analysis.

SSD for each species was calculated using the Lovich–Gibbons index (Lovich and Gibbons 1992)^68^, as proposed by Smith (1999)^69^. The formulas of Lovich–Gibbons index was displayed as follows:

$$\begin{array}{c}{\rm{if}}\,{\rm{females}}\,{\rm{are}}\,{\rm{larger}}:{\rm{SSD}}=({\rm{larger}}\,{\rm{sex}}/{\rm{smaller}}\,{\rm{sex}})\,\mbox{--}\,1\\ {\rm{if}}\,{\rm{males}}\,{\rm{are}}\,{\rm{larger}}:{\rm{SSD}}=-(({\rm{larger}}\,{\rm{sex}}/{\rm{smaller}}\,{\rm{sex}})\,\mbox{--}\,1)\end{array}$$

To compare the relative effects of sample size reduction and the direction of SSD, four datasets were analyzed: (1) a full dataset for which taxonomic inclusion is maximized (45 species for forearm length; 34 species for body mass); (2) a reduced dataset only with taxa with body size measurements from at least 5 individuals of each sex (33 species for forearm length; 27 species for body mass); (3) a reduced dataset with taxa with female-biased SSD (37 species for forearm length; 27 species for body mass); (4) a reduced dataset for species with female-biased SSD and body size measurements from at least 5 individuals of each sex (28 species for forearm length; 17 species for body mass).

Additionally, we collected and analyzed information about habitat types based on the published literature and our own data (Table 2). In this study, we did not consider the bats’ roosting habitats (caves, mines, buildings, and trees). Thus, habitat type was classified based on nighttime activity and foraging behavior. Horseshoe bats all fly close to the substrate and vegetation regardless of habitat type or their body size^43^, but habitat preferences have been observed in some species (e.g., *R*. *mehelyi* in woodland^44^; *R*. *megaphyllus* in woodland^70^, and *R*. *hipposideros* in linear landscape elements like hedgerows or highly structured open landscapes^71^). Here habitat type was classified as open (relative open and less cluttered habitats than forest) or forest (highly cluttered habitats), two types in which Rhinolophdae species can be found. The habitats of 14 bat species (*R*. *capensis*, *R*. *denti*, *R*. *simulator*, *R*. *swinnyi*, *R*. *euryale*, *R*. *arcuatus*, *R*. *clivosus*, *R*. *darlingi*, *R*. *ferrumequinum*, *R*. *fumigatus*, *R*. *hildebrandtii*, *R*. *hipposideros*, *R*. *blasii*, and *R*. *landeri*) were relatively open. These included fynbos, arid areas, savanna woodland, hedgerows, riparian forest, pastures, hedges, and ditches. Most of the 14 species are distributed in Africa and thus occupy relatively open ecosystems such as Savanna^72^. The closed habitats including different types of forest, such as evergreen forest, deciduous forest, rainforests, secondary forest, bamboo forest, and so forth, were home to all other species except for *R*. *euryotis*, *R*. *eloquens*, *R*. *xinzhongguoensis*, *R*. *yunanensis*, *R*. *pumilus*, *R*. *shortridgei*, and *R*. *rouxii* (Table 2). Most of these species were mainly distributed in southeastern Asia and southern China, and so occupy ecosystems with relatively dense vegetation (e.g., forests)^42^. Moreover, many common species (e.g. *R*. *affinis*, *R*. *pearsonii*, *R*. *macrotis*, *R*. *rex*, *R*. *huanus*, *R*. *lepidus*, *R*. *osgoodi*, *R*. *pusillus*, *R*. *sinicus*, *R*. *thomasi*, and *R*. *luctus*), mainly distributed in China, have been observed to frequently forage in forest by acoustic monitoring (personal observation during fieldworks by Tinglei Jiang and Xiaobin Huang). In a previous study^73^, habitat types have been classified as forest or other (habitat other than forest, e.g., savanna, arid, woodland, and fynbos) to assess the contribution of habitat types to echolocation frequency by Bayes Discrete analysis. Differences in wing parameters in bats can lead to various degrees of flexibility in using open space or clutter habitat at both the intraspecific and interspecific levels^74,75^. So far, forage habitat use of many horseshoe bats is still unknown. In light of facts, we here considered it appropriate to classify the habitat types of horseshoe bats as open or forested, because moderately cluttered intermediate habitats (between forest and open) were difficult to define for horseshoe bats in the absence of accurate data regarding habitat use.

We also collected male baculum length and width in horseshoe bats based on the published literature and our own data (Table 2). We obtained data about male baculum length and width from 20 horseshoe bats in this study. Although the mating system is very important to explain the evolution of SSD, we did not collected this data because little is known about it.

### Statistical Analysis

All variables were log_10_-transformed, and we performed tests of normality using the Kolmogorov–Smirnov test with Lilliefors correction. The results showed that all variables met assumptions of a normal distribution (*P* > 0.05, Table 1). We calculated the allometric slope as the reduced major axis regression of log_10_ (male size) on log_10_ (female size) and tested whether the slope was significantly different from 1. Some authors suggested placing males to the *x*-axis when SSD is assessed based upon a log/log plot of the size of one sex against the size of the other sex^8,76^, but the other authors have preferred to assign females on the *x*-axis^25,77^. Thus, there is still no convention to assign of the sexes on the x- and y-axe^78^. Here we place female size on this *x*-axis when we estimated SSD from a log/log plot by the reduced major axis regression of log_10_ (male size) on log_10_ (female size). In this case, the slope >1 indicated allometry consistent with Rensch’s rule. We used the Smart R package^79^ for these analyses. A general linear regression model was used to examine the relationship between SSD and baculum size using an identity link function and a gaussian error structure, and ANOVA was used for testing the effect of habitat types on SSD.

### Phylogeny

Although nuclear introns may exceed mitochondrial DNA in interspecific phylogenetic reconstruction, mitochondrial DNA remains a very useful marker for studying phenotypic evolution because the mtDNA phylogeny can quickly and cheaply provides a global overview of the phylogenetic relationships^48,80^. Moreover, mtDNA sequences on online databases (e.g., GenBank) were more complete than nuclear markers in both within and between species. In this study, we first checked the GenBank and found that a large number of *cytb* gene sequences rather than the other molecular markers were already available for a large proportion of horseshoe bats. For these reasons, we here used *cytb* gene sequence data to reconstruct the phylogeny. Two closely related species (*Hipposideros armiger* and *H*. *cineraceus*) were used to root the tree. We obtained *cytb* sequences from Genbank (see Table 2 for accession numbers). Sequences were aligned with ClustalW^81^. After visual inspection, they were imported into jModelTest 0.1.1^82^ to calculate the best-fit model of nucleotide substitution for the *cytb* gene according to Akaike information criterion (AIC). The most complex general-time-reversible model (GTR + I + *Γ*) was chosen as the best substitution model for this gene. Maximum likelihood (ML) tree reconstruction was conducted in PAUP* 4.0 and RaxML. We then used the R-package ape^83^ to prune species for which we had no morphological or ecological data (analyses including forearm length: N = 38, Fig. 1a; analyses involving body mass: N = 32, Fig. 1b; analyses involving baculum length: N = 19; analyses including habitat type: N = 30).

### Ancestral Size Reconstruction

We reconstructed ancestral states of continuous characters (male and female size, and SSD) on the ML tree pruned for outgroups and species without forearm length data using parsimony analyses in Mesquite version 2.75. We sought to understand evolutionary changes of characters rather than the probability of particular ancestral states on the phylogeny.

### Phylogenetic Comparative Analyses

We also performed phylogenetic reduced major axis regressions^84^ using the phyl.RMA function in the phytol package to estimate phylogenetically informed allometric slopes for 38 species that had *cytb* sequence information.

We measured the strength of the phylogenetic signal in our continuous variables (male and female forearm length, male and female body mass, SSD, baculum size) by estimating Pagel’s λ^85,86^ and Blomberg’s K^87^ using the phytools package^84^. In addition, we tested whether estimates of these two metrics of phylogenetic signal were significantly different from 0 (no phylogenetic signal).

Then we used phylogenetic generalized least squares (PGLS)^86,88^ to test for a relationship between 1) male and female body mass, 2) SSD in body mass and male or female body mass, 3) SSD and the baculum length. Finally, since SSD in forearm length was normally distributed, we used phylogenetic analyses of variance (ANOVA) to assess whether SSD was influenced by habitat types (open habitats, closed habitats). Phylanovas (10,000 iterations) were conducted using the geiger package^89^. In these analysis, SSD was calculated using the Lovich–Gibbons index based on body mass and forearm length. All statistical analyses were carried out in R^90^.

### Ethics Statement

Our work did not cause any physical injuries to bats. All research involving animals was carried out in accordance with the relevant laws for experiments involving vertebrates of the People’s Republic of China, and approved by the National Animal.

Research Authority in Northeast Normal University, China ((Permit Number: NENU-W-2008–108).

### Data availability

The datasets generated and/or analysed during the current study are available from the corresponding author on reasonable request.
